# Memory type Bayesian adaptive max-EWMA control chart for weibull processes

**DOI:** 10.1038/s41598-024-59680-6

**Published:** 2024-04-18

**Authors:** Abdullah A. Zaagan, Imad Khan, Amel Ayari-Akkari, Aamir Raza, Bakhtiyar Ahmad

**Affiliations:** 1 Department of Mathematics, Faculty of Science, Jazan University, P.O. Box 2097, 45142 Jazan, Saudi Arabia; 2 Department of Statistics, Abdul Wali Khan University Mardan, Mardan, Pakistan; 3 Biology Department, College of Sciences in Abha, King Khalid University, P.O. Box 960, Abha, Saudi Arabia; 4 Government College Women University, Sialkot, Pakistan; 5 Higher Education Department, Kabul, Afghanistan

**Keywords:** Average run length, Bayesian approach, Control chart, Inverse response function, Max-EWMA, The Weibull process, Engineering, Mathematics and computing, Statistics

## Abstract

The simultaneous monitoring of both the process mean and dispersion has gained considerable attention in statistical process control, especially when the process follows the normal distribution. This paper introduces a novel Bayesian adaptive maximum exponentially weighted moving average (Max-EWMA) control chart, designed to jointly monitor the mean and dispersion of a non-normal process. This is achieved through the utilization of the inverse response function, particularly suitable for processes conforming to a Weibull distribution. To assess the effectiveness of the proposed control chart, we employed the average run length (ARL) and the standard deviation of run length (SDRL). Subsequently, we compared the performance of our proposed control chart with that of an existing Max-EWMA control chart. Our findings suggest that the proposed control chart demonstrates a higher level of sensitivity in detecting out-of-control signals. Finally, to illustrate the effectiveness of our Bayesian Max-EWMA control chart under various Loss Functions (LFs) for a Weibull process, we present a practical case study focusing on the hard-bake process in the semiconductor manufacturing industry. This case study highlights the adaptability of the chart to different scenarios. Our results provide compelling evidence of the exceptional performance of the suggested control chart in rapidly detecting out-of-control signals during the hard-bake process, thereby significantly contributing to the improvement of process monitoring and quality control.

## Introduction

Statistical process control (SPC) is an essential quality management approach ensuring efficient operations and consistent high-quality products and services. It employs statistical methods such as control charts and process capability analysis to detect and rectify production variations, fostering data-driven decision-making, process stability, and waste reduction. SPC implementation promotes proactive quality management, resulting in enhanced customer satisfaction, cost reduction, and improved operational efficiency. SPC serves as a systematic approach for managing and enhancing the quality of production processes. It primarily utilizes control charts (CCs), graphically depicting process performance by tracking data points over time. SPC aids in recognizing patterns and fluctuations, distinguishing between natural variability (common causes) and irregularities caused by specific events (special causes). By enabling early identification of process deviations, CCs empower organizations to implement timely corrective measures, ensuring consistent adherence to predefined quality standards. As a fundamental quality management tool, SPC fosters continuous improvement and operational efficiency across various industries. In the genesis of control charts (CCs), Shewhart^1^ laid the foundation, employing current sample data to identify significant changes in production processes. In contrast, memory-type CCs, exemplified by cumulative sum (CUSUM) and exponentially weighted moving average (EWMA) CCs, introduced by Page^2^ and Roberts^3^, respectively, incorporate both current and historical sample data. Notably, CUSUM and EWMA CCs exhibit heightened sensitivity in detecting subtle to moderate shifts in process parameters, surpassing the capabilities of traditional Shewhart CCs. These memory-type CCs, especially CUSUM and EWMA, find extensive application across diverse domains, prominently in chemical and industrial production processes. You et al.^4^ evaluate the performance of the EWMA median chart using EARL as a metric and show consistent performance with both EARL and ARL metrics when the deterministic shift size matches the process layer size range. Chatterjeea et al.^5^ introduces the TEWMA-Max CC, demonstrating its higher sensitivity compared to Max-EWMA and Max-DEWMA in detecting shifts in mean and variability, with a satisfactory overall performance for various shift combinations. Jalilibal et al.^6^ examines the need for continuous process monitoring emphasizing the importance of detecting changes in dispersion and location parameters of process. Yung^7^ highlights green manufacturing policies to minimize waste, emphasizing optimal product quality through the Qpm MQCAC for joint monitoring of mean µ and standard deviation σ, demonstrating its efficacy in quality improvement and aligning with environmental goals. Arif et al.^8^ proposed a new control chart integrating EWMA with the generalized likelihood ratio test statistic for joint monitoring of process mean and dispersion under double ranked set sampling. Noor-ul-Amin et al.^9^ introduced a Max-EWMA control chart for joint monitoring of mean and dispersion under the Weibull distribution, showcasing improved sensitivity compared to the existing Max-EWMA control chart. Evaluation is based on ARL and SDRL, with practical application demonstrated through two examples. Lee and Lin^10^ integrate adaptive techniques with Max charts for enhanced process control, utilizing a Markov chain approach to develop statistical indicators and design models. Comparing the adaptive Max charts with EWMA, CUSUM, and double-sampling charts, it demonstrates their superior ability to detect small shifts in mean and variance. Nazir et al.^11^ studied four robust adaptive EWMA schemes for monitoring process location parameters, examining their performance under normal and contaminated environments through comparisons of ARL, SDRL, and run length distribution percentiles, with practical examples included for implementation. Sarwar and Noor-ul-Amin^12^ propose an adaptive EWMA chart for monitoring the mean of a normal process, showcasing its efficacy in detecting shifts through comprehensive Monte Carlo simulations. Noor-ul-Amin et al. ^9^ introduces a CC for jointly identifying the variations in the process mean and dispersion, with the help of inverse response function for Weibull distribution. Previous studies indicate a prevalent dependence on traditional methods centered solely on sample data, often overlooking prior information. Conversely, the Bayesian methodology incorporates both sample data and prior knowledge to revise and construct a posterior distribution (P), thereby improving the estimation process. Menzefricke^13^ presents combined EWMA charts for mean and variance using Bayesian approach. Simulations compare charts for different specifications, including weighing constant and calibration sample size, and evaluate the variance-focused EWMA chart's performance. Aunali and Venkatesan^14^ suggested a Bayesian estimator for quick detection of small shifts in a CC assuming a normal process. It compares this estimator to classical Moving Average, CUSUM, and EWMA control charts in detecting small shifts in the process mean, utilizing a simulation study based on individual observations. Alia^15^ Bayesian predictive monitoring of time-between-events using CUSUM and EWMA CCs with predictive control limits, demonstrating feasibility for online process monitoring and comparison with frequentist sequential charts. Asalam and Anwar^16^ introduce a Bayesian Modified-EWMA chart, indicating higher performance in monitoring slight to large changes and surpassing current alternatives. Bourazasa et al.^17^ propose a versatile Bayesian method for online monitoring using power priors for outlier detection and accommodating diverse data distributions, showcasing superiority over frequentist approaches in an extended simulation study, with practical applications in short production runs and online Phase I monitoring. Khan et al.^18^ proposed a Bayesian HEWMA control chart utilizing RSS schemes, demonstrating improved sensitivity for detecting out-of-control signals compared to existing Bayesian control charts, as evidenced by extensive Monte Carlo simulations and a numerical example from semiconductor manufacturing. Noor-ul-Amin et al.^19^ introduce a novel Bayesian Max-EWMA control chart for simultaneous tracking of non-normal process mean and dispersion, demonstrating superior sensitivity and adaptability under different LFs for Weibull processes, particularly in semiconductor manufacturing. Iqbal et al.^20^ introduce a novel Bayesian Max-EWMA control chart for concurrent monitoring of mean and variance under various loss functions, demonstrating superior sensitivity and performance compared to existing methods. This is validated through Monte Carlo simulations and a practical case study in semiconductor manufacturing. Differing from prior studies exploring classical and Bayesian methods, as well as SRS and RSS schemes, for monitoring mean, variance, and their joint behavior, this article focuses uniquely on the use of Bayesian approaches for monitoring both mean and variance by adapting the value of smoothing constant that helps to adjust the value of smoothing constant according to the estimated shift size. This adaptation ensures that the monitoring process remains effective across a wide range of shift sizes, a capability not addressed in prior studies. The performance evaluation of this Bayesian Max-EWMA CC is carried out through the use of Monte Carlo simulations. The article is structured with specific segments, including a part dedicated to Bayesian theory and various LFs in Section "Bayesian approach", the proposed Bayesian adaptive Max-EWMA CC method in Section "Suggested Bayesian adaptive max-EWMA CC for Weibull process", Section "Simulation study" contains simulation study and extensive discussions and significant findings in Section "Results discussion and main findings", practical applications using real-life data in Section "Real Life application", and concluding remarks in Section "Conclusion".

## Bayesian approach

The Bayesian methodology in statistics sights probability as an indication of the plausibility or confidence in an event. It employs Bayes' theorem to revise the probability associated with a hypothesis in response to additional evidence. In contrast to frequentist statistics, which regard probability as a frequency-based limit, the Bayesian approach integrates prior knowledge or assumptions about relevant parameters during analysis. By merging prior information with fresh data, it facilitates the determination of unknown parameters and the measurement of uncertainty in a more adaptable and intuitive manner. This approach is widely applicable across a range of fields, including machine learning, decision-making, and experimental design. Let V be the random variable representing lifetimes following a Weibull distribution with both the shape parameter (λ) and scale parameter (α) being greater than zero (α > 0, λ > 0). In this scenario, the mathematical expressions for the probability density function (pdf) and cumulative distribution function (cdf) are as follows:

1 $$ f\left( {v,\alpha ,\lambda } \right) = \frac{{\lambda v^{{\left( {\lambda - 1} \right)}} }}{{\alpha^{\lambda } }}\exp \left( { - \left( {\frac{v}{\alpha }} \right)^{\lambda } } \right);v > 0, $$

2 $$ F\left( v \right) = \Pr \left( {V \le v} \right) = 1 - \exp \left( { - \left( {\frac{v}{\alpha }} \right)^{\lambda } } \right);v > 0 $$

### Squared error loss function

The SELF is a critical tool in the Bayesian framework, used to gauge the gap between predicted and actual parameter values. It computes the average squared difference between estimated and true parameter values, assessing the model's suitability. In Bayesian decision theory, it aids in choosing an optimal estimator that minimizes the expected value of the squared error loss. Additionally, this function plays a key role in Bayesian inference, helping to derive posterior distributions and evaluate model accuracy. The SELF, originally presented by Gauss^21^, consider the interest as *X* and estimator $\hat{\theta }$ employed for the estimation of population parameter, referred to as θ. Its expression for SELF is mathematized as:

3 $$ L\left( {\theta ,\hat{\theta }} \right) = \left( {\theta - \hat{\theta }} \right)^{2} $$

Applying SELF, Bayes estimator is mathematized as

4 $$ \hat{\theta }_{{\left( {SELF} \right)}} = E_{\theta /x} \left( \theta \right) $$

### Linex loss function

In Bayesian methodology, the linex loss function (LLF) is instrumental in gauging the difference between the estimated and true values of a parameter. It measures the absolute difference between these values, allowing for an evaluation of the model's accuracy and reliability. This function aids in the selection of an appropriate estimator to minimize the expected value of the LLF within Bayesian decision theory. Furthermore, it contributes to Bayesian inference by helping derive posterior distributions and improving the assessment of model performance and precision. Varian^22^ introduced the LLF to minimize risks in Bayesian estimation. The mathematical representation of the LLF is as follows:

5 $$ L\left( {\theta ,\hat{\theta }} \right) = \left( {e^{{c\left( {\theta - \hat{\theta }} \right)}} - c\left( {\theta - \hat{\theta }} \right) - 1} \right) $$

Bayesian estimator $\hat{\theta }$ under LLF is mathematizied as

6 $$ \hat{\theta }_{{\left( {LLF} \right)}} = - \frac{1}{c}InE_{\theta /x} \left( {e^{ - c\theta } } \right) $$

## Suggested Bayesian adaptive max-EWMA CC for Weibull process

In the context of statistical analysis, let's examine a sequence of random samples represented as V_i1_, V_i2_, …, V_in_, derived from a Weibull distribution represented at various time instances, with i representing distinct sequences ($i = \, 1, \, 2, \, 3$, and so forth). It is common for Weibull process that the parameters ($\alpha$ and $\lambda$) to be initially unknown, prompting the need for estimation using historical data. The process of parameter estimation typically assumes the process is under control. Let $\alpha_{0}$ and $\lambda_{0}$ represent the estimated scale and shape parameters, respectively. The estimation of these parameters involves establishing a connection between standard normal and Weibull distribution, as elucidated Faraz et al.^23^ in Eq. (7), expressed as:

7 $$ \phi \left( {z,\alpha ,\lambda } \right) = f\left( {W^{ - 1} \left( {z,\alpha ,\lambda } \right)} \right)\frac{dt}{{dz}} $$

The properties of the above function is given as

8 $$ \mu_{z} = E\left( {Z,\alpha ,\lambda } \right) = \int z \phi \left( {z,\alpha ,\lambda } \right)dz $$

and

9 $$ \delta_{z}^{2} = Var\left( {Z,\alpha ,\lambda } \right) = \int {\left( {z - \mu } \right)^{2} } \phi \left( {z,\alpha ,\lambda } \right)dz $$

Useful information about the effects of changing the parameters of a Weibull distribution on the mean and variance of a random variable with a standard normal distribution can be obtained from Eqs. (8) and (9). Essentially, these formulas express the precise influence that changes in the Weibull distribution parameters have on the features and attributes of the normal distribution.

Suppose we have a series of random samples, denoted as $z_{i1} = W\left( {V_{i1} ,\alpha_{0} ,\lambda_{0} } \right)$, $z_{i2} = W_{N} \left( {V_{i2} ,\alpha_{0} ,\lambda_{0} } \right)$, …, $z_{in} = W_{N} \left( {V_{in} ,\alpha_{0} ,\lambda_{0} } \right)$, originating from a Weibull distribution and transformed to a normal distribution. In this context, the Max-EWMA Control Chart, integrating the principles of Bayesian theory, allows for the concurrent monitoring of both the mean and variance of the process that follows the normal distribution. This approach facilitates effective and simultaneous monitoring of the underlying process parameters, ensuring improved control and management of the system's performance.

10 $$ f\left( {z_{t} :\theta ,\sigma^{2} } \right) = \frac{1}{{\sqrt {2\pi \sigma^{2} } }}\exp \left( { - \tfrac{1}{{2\sigma^{2} }}\left( {z_{t} - \theta } \right)^{2} } \right) $$

In a Bayesian framework, assuming normal distributions for both the likelihood function and prior distribution, the resulting posterior distribution also adopts a normal distribution form. This distribution is characterized by its mean (θ) and variance (σ) as defining parameters. The probability density function (pdf) of the posterior distribution is articulated as:

11 $$ P\left( {\theta /z} \right) = \frac{1}{{\sqrt {2\pi } \sqrt {\frac{{\delta^{2} \delta_{0}^{2} }}{{\delta^{2} + n\delta_{0}^{2} }}} }}\exp \left[ { - \frac{1}{2}\left( {\frac{{\theta - \sum\limits_{i = 1}^{n} {\frac{{z_{i} \delta_{0}^{2} + \theta_{0} \delta_{0}^{2} }}{{\delta^{2} + n\delta_{0}^{2} }}} }}{{\sqrt {\frac{{\delta^{2} \delta_{0}^{2} }}{{\delta^{2} + n\delta_{0}^{2} }}} }}} \right)^{2} } \right] $$

where $\theta_{n} = \frac{{n\overline{z} \delta_{0}^{2} + \delta^{2} \theta_{0} }}{{\delta^{2} + n\delta_{0}^{2} }}$ and $\delta_{n}^{2} = \frac{{\delta^{2} \delta_{0}^{2} }}{{\delta^{2} + n\delta_{0}^{2} }}$ respectively.

Selecting a sample of “*n*” values that represent a specific quality attribute “*Z*” from the production process is the first step in creating a Max-EWMA chart using Bayesian methodology. Then, using the SELF method, we calculate the transformed statistics for the mean and variance as shown below:

12 $$ U_{t} = \frac{{\hat{\theta }_{(SELF)} - \theta }}{{{\raise0.7ex\hbox{$\delta $} \!\mathord{\left/ {\vphantom {\delta {\sqrt n }}}\right.\kern-0pt} \!\lower0.7ex\hbox{${\sqrt n }$}}}} $$

and

13 $$ V_{t} = \phi^{ - 1} \left[ {H\left\{ {\frac{{\left( {n - 1} \right)\hat{\delta }_{(SELF)}^{2} }}{{\delta^{2} }}} \right\},\left( {n - 1} \right)} \right] $$

where $\hat{\theta }_{(SELF)} = \frac{{n\overline{x} \delta_{0}^{2} + \delta^{2} \theta_{0} }}{{\delta^{2} + n\delta_{0}^{2} }}$ and $\hat{\delta }_{(SELF)}^{2} = \frac{{\delta^{2} \delta_{0}^{2} }}{{\delta^{2} + n\delta_{0}^{2} }}$ are the Bayes estimators applying SELF, while using LLF, the Bayes estimators are given as $\hat{\theta }_{{\left( {_{LLF} } \right)}} = \frac{{n\overline{z} \delta_{0}^{2} + \delta^{2} \theta_{0} }}{{\delta^{2} + n\delta_{0}^{2} }} - \frac{{C{\prime} }}{2}\delta_{n}^{2}$ and $\hat{\delta }_{{\left( {LLF} \right)}}^{2} = \frac{{\delta^{2} \delta_{0}^{2} }}{{\delta^{2} + n\delta_{0}^{2} }}$, The mathematical description of the transformed statistic for both the process mean and variance under LLF is as follows:

14 $$ U_{t} = \frac{{\hat{\theta }_{(LLF)} - \theta }}{{{\raise0.7ex\hbox{$\delta $} \!\mathord{\left/ {\vphantom {\delta {\sqrt n }}}\right.\kern-0pt} \!\lower0.7ex\hbox{${\sqrt n }$}}}} $$

and

15 $$ V_{t} = \phi^{ - 1} \left[ {H\left\{ {\frac{{\left( {n - 1} \right)\hat{\delta }_{(LLF)}^{2} }}{{\delta^{2} }}} \right\},\left( {n - 1} \right)} \right] $$

In this context, $H\left( {n,\nu } \right)$ represents a chi-square distribution with $\nu$ degrees of freedom, and $\phi^{ - 1}$ signifies the inverse function. The following is the breakdown of the calculations for the EWMA statistics related to the process mean and dispersion.

16 $$ P_{t(LF)} = \lambda U_{t(LF)} + \left( {1 - \lambda } \right)P_{t - 1(LF)} $$

17 $$ Q_{t(LF)} = \lambda V_{t(LF)} + \left( {1 - \lambda } \right)V_{t - 1(LF)} $$

In this scenario, for the EWMA statistic Pt and Qt, the initially values are denoted by $P_{0}$ and $Q_{0}$ respectively. Furthermore, both Pt and Qt are independent and normally distributed with a mean of zero and variances of σ^2^ respectively. This is expressed as follows:

### Integration of adaptive approach

Subsequently, we introduce the adaptive technique. Consider ${X}_{t}$ a normally distributed random variable at time *t* with a sample size of *n*, characterized by a mean ${\upmu }_{{\text{X}}}$ and a variance ${\upsigma }_{{\text{X}}}^{2}.\mathrm{ i}.{\text{e}}.,{{\text{X}}}_{{\text{t}}}\sim {\text{N}}\left({\upmu }_{{\text{X}}}, {\upsigma }_{{\text{X}}}^{2}\right)$. Jiang et al.^24^ introduced an estimator for estimating the magnitude of the shift using the following expression:

18 $$ \hat{\delta }_{t}^{*} = \psi X_{t} + \left( {1 - \psi } \right)\hat{\delta }_{t - 1}^{*} $$

The symbol ψ represents the smoothing constant, typically constrained to the range (0,1]. In real-world applications, having prior knowledge about the precise magnitude of a shift is uncommon. Consequently, we initially estimate its value. Haq et al.^25^ suggested an unbiased estimator, denoted as $$\hat{\delta }_{t}^{{**}}$$, which can be expressed as:

19 $$ \hat{\delta }_{t}^{*} = \frac{{\hat{\delta }_{t}^{*} }}{{1 - (1 - \psi )^{t} }} $$

where $${\text{E}}\left( {\hat{\delta }_{t}^{{**}} } \right) = {\updelta } = 0$$. The process remains stable for a certain period of time, denoted as $${\text{t}}\le {{\text{t}}}_{0}$$. In practical scenarios, the true magnitude is frequently unknown. Instead, δ is estimated by considering $$\tilde{\delta }_{t}^{{**}} = \left| {\hat{\delta }_{t}^{{**}} } \right|$$.

The detection is accomplished through the recursive calculation of the subsequent EWMA statistic, known as the suggested chart statistic, as follows:

20 $${A}_{t}=\upeta ({\tilde{\upomega }}_{t}^{*}){X}_{t}+\left(1-\upeta ({\tilde{\upomega }}_{t}^{*})\right){A}_{t-1}$$

Setting the initializing value as ${{\text{A}}}_{0}$ = 0, we avoid using a fixed value for the smoothing constant. Instead, we adopt a function that can adjust to different values depending on the changing conditions of the process. To achieve this, we propose the following function for the self-adjusting weighting factor η(${\tilde{\upomega }}_{{\text{t}}}^{*}$).

21 $$\eta ({\tilde{\omega }}_{t}^{*})=\left\{\begin{array}{c}\begin{array}{ccc} \frac{{{\tilde{\delta }}_{t}}^{2}}{7\left(1+{{\tilde{\delta }}_{t}}^{2}\right)}& \text{ , }& {\tilde{\delta }}_{t}\le 1.0 \end{array}\\ \begin{array}{ccc} \frac{{\tilde{\delta }}_{t}}{7\left(1+{\tilde{\delta }}_{t}\right)}& \text{ , }& 1.0<{\tilde{\delta }}_{t}\le 2.7\\ 1& \text{ , }& {\tilde{\delta }}_{t}> 2.7\end{array}\end{array}\right.$$

The function is segmented into three parts based on the estimated shift. In the initial part, the function is constructed when the estimated shift is less than 1, resulting in a small smoothing constant to accommodate minor shifts. In the second segment, the smoothing constant takes on a larger value compared to the first part, aligning with moderate estimated shifts. For shifts categorized as large, the third part of the function assigns a smoothing constant equal to 1. Here, *η(*${\tilde{\omega }}_{t}^{*}$*)* represents a random variable shaped by a continuous function, optimizing the performance of our chart for shift detection. When ${\tilde{\delta }}_{t}$ ≤ 2.7, *η()* is specifically designed to be sensitive to small to moderate shifts. Our adaptive chart outperforms other methods by excelling in detecting shifts of any size. If the shift surpasses ${\tilde{\delta }}_{t}$ ≥ 2.7, our chart functions similarly to a Shewhart chart, effectively identifying larger shifts with ${\tilde{\delta }}_{t}$= 2.7 serving as the pivotal point. It provides flexibility in model adjustments and the inclusion of additional factors. Instead of using a fixed value in Eqs. (10) and (11), assuming its constancy throughout the process, we utilize the self-adjusting function from Eq. (16) and iteratively update Eqs. (10) and (11) to calculate the individual EWMA under the adaptive approach. Thus, the transformed equations are mathematically described as:

22 $${P}_{t(LF)}=\eta \left({\tilde{\omega }}_{t}^{*}\right){U}_{t\left(LF\right)}+\left(1-\eta \left({\tilde{\omega }}_{t}^{*}\right)\right){P}_{t-1\left(LF\right)},$$

23 $${Q}_{t(LF)}=\eta \left({\tilde{\omega }}_{t}^{*}\right){V}_{t\left(LF\right)}+\left(1-\eta \left({\tilde{\omega }}_{t}^{*}\right)\right){V}_{t-1\left(LF\right)}.$$

Finally, these values are inserted into the Max-EWMA provided by Chen and Change^26^ to jointly monitor the process mean and variance in a single chart. Thus, the plotting can be expressed as:

24 $$ Z_{t} = Max\left( {\left| {P_{t(LF)} } \right|,\left| {Q_{t(LF)} } \right|} \right),\;{\text{for}}\;t = 1,2,.. $$

where Max is function to get the maximum value of the given inputs.

The suggested statistic being a positive value simplifies the monitoring process. In this context, we only need to plot the UCL for the joint monitoring of the process mean and variance. The plotting statistic is then compared with the UCL threshold. If the value is above the UCL, the process is considered out of control, indicating deviations in the mean, variance, or both. On the other hand, if the value is below the UCL, the process is considered to be under control.

## Simulation study

The study employs the Monte Carlo simulation method to intricately examine the effectiveness of the Bayesian adaptive Max-EWMA CC in identifying shifts in process parameters through the computation of ARLs and SDRLs, with a particular focus on the in-control process ARL set at 370. This approach is pivotal for understanding the impact of varying the smoothing constant, such as $\gamma$ = 0.10 and 0.25, the study aims to shed light on how these adjustments can enhance the CCs responsiveness to detecting quality shifts in manufacturing processes. The Monte Carlo simulation method's comprehensive steps are crafted to provide a deep dive into the performance of the Bayesian adaptive Max-EWMA CC, evaluating its potential in improving process monitoring and control. This rigorous analysis not only highlights the adaptability and efficiency of the proposed control chart in signaling out-of-control conditions but also contributes valuable insights into optimizing process quality management strategies, thereby offering a significant advancement in the field of SPC. The following are main steps.

### *Step 1.* setting up control limits

i. We take Weibull distribution as a sampling distribution and transform to standard normal distribution. The prior distribution is also a standard normal for calculating the mean and variance of posterior distribution under different LF.
ii. Chose the initial value of *h* with a specific value of $\gamma$ at fixed ${ARL}_{0}$= 370.
iii. Random samples of size *n* is generated for the in-control process from the normal distribution such that $X\sim N\left( {E(\hat{\theta }),E\left( {\hat{\delta }} \right)} \right)$.
iv. Compute the plotting statistic of the offered Bayesian adaptive Max-EWMA CC and evaluate the process according to the suggested design.
v. If the process declared in-control, then repeat steps (iii-iv) until the process is showed to be out-of-control. When the process is declared as out-of-control then record the in-control number as run length.
vi. Repeat the steps (iii-v) 50,000 times and calculate the in-control ARL.
vii. If ${ARL}_{0}=370$, then go to Step 2 with the same value of $\gamma$, and *h*. Otherwise, repeat steps (ii-vi) with the change value of *L*.

### *Step 2.* Evaluate the out-of-control ARL

i. Select random sample for the shifted process from the normal distribution such that $X\sim N\left( {E(\hat{\theta }) + \sigma \frac{{E\left( {\hat{\delta }} \right)}}{\sqrt n },E\left( {\hat{\delta }} \right)} \right),$ where $\sigma$ is the amount of shift in the process parameter.
ii. Calculate plotting statistic and appraise the process according to the design of the recommended CC.
iii. If the plotted statistic lies within the UCL, repeat steps (i–ii). Otherwise, record the number of generated points, indicating a single out-of-control run length.
iv. Repeat the aforementioned process (steps i–iii) 50,000 times to determine the ARL_1_ and SDRL_1_ for out-of-control scenarios.

## Results discussion and main findings

In this examination, Tables 1, 2, 3 and 4 play a pivotal role in presenting the extensive findings resulting from the implementation suggested CC in the context of the Weibull process. The study conducts a detailed exploration of the implications of two different LFs, highlighting the significance of the posterior distribution. In the context of informative priors, these assessments are conducted by incorporating pre-existing knowledge and beliefs into the analytical procedures. This integration aims to augment the overall understanding of the results, recognizing and leveraging prior information to inform the Bayesian analysis. The use of 50,000 replicates ensures robust statistical findings, enhancing the reliability of the ARL and SDRL calculations. Careful selection of smoothing constants demonstrates a commitment to precision, refining the analysis and enabling a thorough evaluation of the suggested Bayesian CC performance in diverse scenarios. Additionally, the study comprehensively explores a wide range of combinations, spanning from 0.00 to 3.00 of shift values (a) and from 0.25 to 3.00 for variance shift values (b). In this comprehensive study, we thoroughly examine the efficacy of the offered CC designed for jointly identifying both the process mean and dispersion. The study's findings unequivocally highlight the exceptional sensitivity of the method in detecting deviations from established norms within production processes. This underscores the significant potential of the proposed CC as a reliable tool for continuous monitoring and quality control across various industrial sectors. It is crucial to continuously confirm during the analysis that the plotting statistic was computed stays less than the UCLi. The outcome of each trial is determined by the plotting statistic exceeding the UCLi, which indicates a substantial change in the process mean and standard deviation. These observed changes are associated with variations in the parameters of the Weibull distribution, indicating potential deviations from the expected norms in the production process. In our examination, we specifically investigate shifts in shape parameters within the range of 0.25 to 4.00 and shifts in scale parameters within the range of 0.0 to 5.00. For W(1, 1.5), the process initially adheres to control limits, following a N(0,1) normal distribution. The ARL under in-control conditions was determined as 370 for two distinct values of λ. The findings presented in Tables 1 and 2 robustly affirm the performance of offered CC, particularly when employed in conjunction with SELF applying posterior distribution. This combined approach showcases an impressive capability to detect shifts in both process mean and variance concurrently, ensuring process stability and product quality. Notably, a trend that consistently shows up in the data indicates that ARLs decrease as mean shift magnitude increases. ARLs exhibit a decline in response to variance shifts as well. These consistent patterns strongly emphasize the recommended CC's ability to promptly recognize and indicate process shifts, enabling timely intervention and process control. This characteristic positions it as an invaluable instrument for comprehensive production process monitoring, ensuring the swift identification and resolution of deviations from established norms. The ultimate goal of implementing these control charts is to enhance product quality and improve process efficiency, rendering them valuable assets across diverse industries. For instance, when scrutinizing the ARL results for the suggested Bayesian Max-EWMA CC, emphasis is placed on the application of the SELF. In this context, a smoothing parameter of λ = 0.10 and a sample size of n = 5 are employed. In particular, our exploration involves examining different Weibull distribution shape parameter shift values, spanning from 1.50 to 5.00, while keeping the scale parameter shift constant at 1.0. The corresponding run length outcomes for mentioned shifts are enumerated as follows: 369.15, 80.19, 24.05, 13.71, 10.14, 8.16, 7.12, 4.88, and 4.13. Notably, as shift magnitude increases, a distinct reduction in the corresponding ARL values is observed. This finding underscores the heightened efficiency of the proposed Bayesian Max-EWMA CC in promptly identifying shifts in the shape parameter. Practically, this increased sensitivity implies that the control chart can swiftly identifying minor variations in the process mean, facilitating a rapid response—a critical aspect in upholding process quality and consistency. The analysis extends to evaluating the influence of varying scale parameter values, ranging from 1.0 to 4.00, while keeping the shape parameter constant at a = 1.50. The resultant run length are: 369.51, 24.90, 16.82, 7.16, 5.08, 3.60, 2.60, 2.11, and 1.50. Notably, these results exhibit a distinct trend—as the shape parameter diverges from its baseline value of 1, there is a noticeable decrease in ARL values. This pattern demonstrates how the recommended Control Chart can effectively identify changes in process dispersion quickly. It is crucial to underscore a key observation from Table 2. In assessing the performance of the recommended CC, it becomes apparent that the effectiveness of the CC diminishes with higher values of the smoothing constant. According to this realization, there are some situations where choosing a smaller smoothing constant might be more beneficial for getting the best results. ARL results for the proposed CC using the LLF with a consistent λ = 0.25 and n = 5 are similarly presented in Tables 3 and 4. 370.09, 20.34, 6.34, 2.81, 1.99, 1.57, and 1.17 were The ARL values that resulted from several trials that involved shifting the shape parameter from 1.50 to 5.00 and corresponding shifts in the scale parameter kept at 1.0. The key findings indicate a noticeable pattern: as process shifts become more significant, ARL values show a steep decrease, underlining the remarkable accuracy of the provided control chart in quickly detecting deviations in both process mean and dispersion. It is also important to recognize that the effectiveness of the suggested CC in concurrently identifying process mean and dispersion is impacted by the sample size. The results consistently show that as the sample size grows, ARL values decrease, demonstrating the improved precision of the recommended control chart in promptly identifying deviations from the anticipated process parameters:

- The analysis of the ARL and SDRL across Tables 1, 2, 3 and 4 associated with proposed CC using Weibull process highlights its effectiveness in identifying both process mean and variance, particularly in the detection of minor to moderate shifts. These profiles demonstrate the CC's performance across various scenarios, with a consistent trend indicating its proficiency in swiftly identifying variations in process mean and dispersion, thus establishing its significance and consistency.
- Simulation outcomes clearly show reducing the smoothing constant enhances effectiveness of the recommended Bayesian CC for simultaneous process monitoring. Essentially, decreasing the $\lambda$ makes CC more responsive and adept at swiftly identifying changes in both the process mean and variance. This insight suggests that, in particular situations or applications, opting for a lower $\lambda$ can result in more streamlined monitoring, leading to faster identification of deviations from expected process parameters and ultimately improving the quality and reliability of the process.
- In our investigation, a critical aspect that we have thoroughly examined is the variation in sample size. Our analysis reveals a crucial and compelling insight. Clearly, the efficacy and performance of the proposed CC improve noticeably and significantly with increasing sample size. In practical terms, this suggests that when utilizing larger sample sizes, the CC becomes more skilled at accurately and swiftly detecting changes in both the process mean and variance. This development holds particular significance as it has the potential to improve process monitoring's dependability and resilience, thereby leading to an overall improvement in process consistency and quality.

**Table 1:** ARL and SDRL results of offered CC with *W (1,1.5)* with $\gamma$=0.10 with the sizes 3, 5, and 7.

*α*	*n*	Shape parameter = *λ*											
		0.50	1.00	1.25	1.50	1.75	2.00	2.25	2.50	2.75	3.00	4.00	5.00
		**ARL** **(SDRL)**	**ARL** **(SDRL)**	**ARL** **(SDRL)**	**ARL** **(SDRL)**	**ARL** **(SDRL)**	**ARL** **(SDRL)**	**ARL** **(SDRL)**	**ARL** **(SDRL)**	**ARL** **(SDRL)**	**ARL** **(SDRL)**	**ARL** **(SDRL)**	**ARL** **(SDRL)**
0.25	**(µ,σ)**	**(-1.51,2.04)**	**(-1.49,0.89)**	**(-1.45,0.72)**	**(-1.42,0.61)**	**(-1.40,0.53)**	**(-1.38,0.47)**	**(-1.36,0.42)**	**(-1.35,0.38)**	**(-1.33,0.35)**	**(-1.32,0.32)**	**(-1.29,0.25)**	**(-1.28,0.20)**
	3	1.68 (0.92)	1.85 (0.68)	1.89 (0.58)	1.93 (0.50)	1.95 (0.44)	1.99 (0.38)	2.00 (0.33)	2.01 (0.28)	2.02 (0.25)	2.01 (0.23)	2.00 (0.19)	1.98 (0.17)
	5	1.37 (0.60)	1.48 (0.53)	1.50 (0.51)	1.54 (0.49)	1.56 (0.49)	1.61 (0.48)	1.66 (0.47)	1.64 (0.53)	1.75 (0.42)	1.77 (0.41)	1.82 (0.37)	1.76 (0.42)
	7	1.17 (0.41)	1.16 (0.37)	1.15 (0.35)	1.13 (0.33)	1.11 (0.32)	1.10 (0.31)	1.10 (0.30)	1.08 (0.27)	1.08 (0.27)	1.07 (0.26)	1.03 (0.19)	1.00 (0.09)
0.50	**(µ,σ)**	**(-0.68,3.16)**	**(-0.87,1.11)**	**(-0.84,0.90)**	**(-0.81,0.76)**	**(-0.78,0.66)**	**(-0.76,0.58)**	**(-0.74,0.53)**	**(-0.72,0.48)**	**(-0.71,0.44)**	**(-0.70,0.40)**	**(-0.66,0.31)**	**(-0.64,0.25)**
	3	1.43 (0.73)	3.68 (2.17)	3.86 (1.95)	3.97 (1.82)	4.12 (1.71)	4.13 (1.54)	4.19 (1.49)	4.17 (1.41)	4.04 (1.30)	3.89 (1.22)	3.31 (1.00)	2.83 (0.79)
	5	1.17 (0.41)	2.77 (1.32)	2.85 (1.15)	2.92 (1.03)	2.98 (0.95)	2.96 (0.83)	2.95 (0.78)	2.86 (0.72)	2.74 (0.69)	2.60 (0.64)	2.18 (0.51)	1.93 (0.38)
	7	1.06 (0.25)	2.26 (1.05)	2.35 (0.92)	2.40 (0.83)	2.46 (0.75)	2.45 (0.67)	2.41 (0.63)	2.34 (0.61)	2.19 (0.58)	2.06 (0.55)	1.69 (0.49)	1.40 (0.49)
0.75	**(µ,σ)**	**(0.01,4.18)**	**(-0.42,1.30)**	**(-0.39,1.05)**	**(-0.36, 0.88)**	**(-0.34,0.77)**	**(-0.32, 0.68)**	**(-0.29, 0.61)**	**(-0.28,0.56)**	**(-0.26,0.51)**	**(-0.25,0.47)**	**(-0.21,0.37)**	**(-0.18,0.30)**
	3	1.19 (0.46)	8.01 (7.21)	13.75 (11.24)	16.88 (12.04)	17.08 (11.28)	14.78 (9.28)	11.56 (6.89)	9.33 (5.38)	7.40 (3.88)	6.17 (3.05)	4.18 (1.61)	3.27 (1.07)
	5	1.06 (0.24)	5.51 (4.00)	4.59 (3.21)	10.19 (9.21)	9.52 (5.71)	7.50 (3.87)	5.76 (2.73)	4.67 (1.99)	3.88 (1.47)	3.37 (1.13)	2.51 (0.69)	2.13 (0.48)
		1.01 (0.11)	4.34 (2.82)	6.91 (4.55)	7.81 (4.62)	6.86 (3.56)	5.45 (2.44)	4.18 (1.67)	3.49 (1.26)	2.95 (0.95)	2.61 (0.81)	1.95 (0.54)	1.65 (0.49)
1.0	**(µ,σ)**	**(0.61,5.00)**	**(-0.05,1.47)**	**(-0.03,1.18)**	**(0.00, 1.00)**	**(0.03,0.87)**	**(0.05,0.77)**	**(0.07,0.69)**	**(0.09,0.63)**	**(0.11,0.58)**	**(0.12,0.54)**	**(0.16,0.41)**	**(0.19,0.34)**
	3	1.11 (0.34)	7.91 (7.55)	36.79 (36.18)	370.33 (403.23)	88.94 (63.20)	36.87 (25.26)	20.22 (13.78)	14.14 (8.95)	10.52 (6.22)	8.56 (5.43)	4.84 (2.13)	3.74 (1.34)
	5	1.03 (0.17)	4.95 (3.68)	21.94 (19.72)	369.94 (386.89)	47.13 (33.64)	17.09 (11.50)	9.36 (5.47)	6.57 (3.43)	5.13 (2.39)	4.33 (1.76)	2.81 (0.83)	2.33 (0.59)
	10	1.00 (0.07)	3.82 (2.48)	15.74 (13.42)	370.21 (398.81)	32.97 (23.57)	11.24 (7.04)	6.43 (3.24)	4.68 (2.03)	3.75 (1.41)	3.24 (1.13)	2.19 (0.63)	1.83 (0.50)
1.50	**(µ,σ)**	**(1.67,6.26)**	**(0.58,1.86)**	**(0.58,1.44)**	**(0.61,1.21)**	**(0.64,1.05)**	**(0.66,0.94)**	**(0.68,0.84)**	**(0.70,0.77)**	**(0.72,0.71)**	**(0.74,0.65)**	**(0.79,0.51)**	**(0.82,0.41)**
	3	1.05 (0.24)	2.89 (2.10)	4.73 (3.79)	5.92 (4.54)	5.86 (4.06)	5.66 (3.47)	5.38 (2.96)	5.05 (2.59)	4.76 (2.20)	4.46 (1.92)	3.80 (1.24)	3.40 (0.92)
	5	1.00 (0.09)	2.15 (1.20)	3.40 (2.11)	4.19 (2.63)	4.15 (2.28)	3.96 (1.95)	3.74 (1.64)	3.53 (1.37)	3.32 (1.17)	3.17 (1.01)	2.74 (0.71)	2.44 (0.56)
	7	1.00 (0.02)	1.19 (0.42)	1.21 (0.43)	3.38 (1.92)	3.41 (1.70)	3.21 (1.43)	3.04 (1.21)	2.87 (1.03)	2.74 (0.90)	2.60 (0.78)	2.25 (0.57)	1.98 (0.46)
2.00	**(µ,σ)**	**(2.57,7.16)**	**(1.16,2.43)**	**(1.11,1.70)**	**(1.13,1.41)**	**(1.15,1.23)**	**(1.18,1.09)**	**(1.20,0.98)**	**(1.23,0.89)**	**(1.25,0.82)**	**(1.26,0.76)**	**(1.32,0.59)**	**(1.36,0.48)**
	3	1.03 (0.99)	1.67 (0.97)	2.33 (1.47)	2.31 (1.44)	2.57 (1.37)	2.49 (1.19)	2.41 (1.02)	2.33 (0.91)	2.26 (0.82)	2.22 (0.73)	2.08 (0.53)	2.01 (0.39)
	5	1.00 (0.06)	1.35 (0.59)	1.82 (0.92)	1.98 (0.94)	2.00 (0.88)	1.95 (0.79)	1.89 (0.70)	1.83 (0.64)	1.78 (0.58)	1.76 (0.55)	1.69 (0.47)	1.65 (0.47)
	7	1.00 (0.01)	1.15 (0.39)	1.51 (0.70)	1.63 (0.76)_	1.64 (0.73)	1.56 (0.65)	1.51 (0.60)	1.45 (0.55)	1.40 (0.52)	1.38 (0.50)	1.23 (0.42)	1.13 (0.34)
3.00	**(µ,σ)**	**(4.00,8.33)**	**(2.35,4.00)**	**(2.08,2.58)**	**(2.03,1.86)**	**(2.04, 1.55)**	**(2.07, 1.37)**	**(2.10, 1.24)**	**(2.12, 1.13)**	**(2.15, 1.04)**	**(2.17, 0.96)**	**(2.24, 0.75)**	**(2.29, 0.61)**
	3	1.01 (0.13)	1.14 (0.38)	1.31 (0.59)	1.41 (0.64)	1.40 (0.61)	1.37 (0.55)	1.34 (0.52)	1.31 (0.49)	1.27 (0.45)	1.25 (0.44)	1.13 (0.34)	1.07 (0.26)
	5	1.00 (0.04)	1.03 (0.19)	1.12 (0.34)	1.19 (0.42)	1.18 (0.40)	1.14 (0.35)	1.12 (0.32)	1.08 (0.28)	1.06 (0.25)	1.04 (0.21)	1.00 (0.09)	1.00 (0.03)
	7	1 (0)	1.00 (0.08)	1.04 (0.21)	1.07 (0.26)	1.05 (0.23)	1.03 (0.19)	1.02 (0.14)	1.01 (0.11)	1.00 (0.07)	1.00 (0.04)	1.00 (0.01)	1 (0)
4.00	**(µ,σ)**	**(5.12,9.07)**	**(3.61,5.53)**	**(3.15,3.99)**	**(2.93,2.81)**	**(2.86, 2.07)**	**(2.86, 1.68)**	**(2.88, 1.48)**	**(2.91, 1.35)**	**(2.94, 1.24)**	**(2.96, 1.15)**	**(3.04, 0.89)**	**(3.10, 0.73)**
	3	1.01 (0.11)	1.04 (0.22)	1.09 (0.31)	1.15 (0.39)	1.15 (0.39)	1.12 (0.34)	1.09 (0.30)	1.06 (0.25)	1.05 (0.22)	1.03 (0.19)	1.00 (0.08)	1.00 (0.02)
	5	1.00 (0.03)	1.00 (0.08)	1.01 (0.13)	1.04 (0.20)	1.04 (0.20)	1.02 (0.15)	1.01 (0.11)	1.00 (0.08)	1.00 (0.06)	1.00 (0.04)	1.0 (0.0)	1.0 (0.0)
	7	1 (0)	1.00 (0.02)	1.00 (0.05)	1.00 (0.09)	1.00 (0.08)	1.00 (0.05)	1.00 (0.02)	1.00 (0.01)	1 (0)	1.00 (0.0)	1.0 (0.0)	1.0 (0.0)

**Table 2:** Run length outcomes for the recommended CC using *W(1,1.5)* with $\gamma$=0.25 and applying sizes of 3, 5, and 7.

*α*	*N*	Shape parameter = *λ*											
		0.50	1.00	1.25	1.50	1.75	2.00	2.25	2.50	2.75	3.00	4.00	5.00
		**ARL** **(SDRL)**	**ARL** **(SDRL)**	**ARL** **(SDRL)**	**ARL** **(SDRL)**	**ARL** **(SDRL)**	**ARL** **(SDRL)**	**ARL** **(SDRL)**	**ARL** **(SDRL)**	**ARL** **(SDRL)**	**ARL** **(SDRL)**	**ARL** **(SDRL)**	**ARL** **(SDRL)**
0.25	**(µ,σ)**	**(-1.51,2.04)**	**(-1.49,0.89)**	**(-1.45,0.72)**	**(-1.42,0.61)**	**(-1.40,0.53)**	**(-1.38,0.47)**	**(-1.36,0.42)**	**(-1.35,0.38)**	**(-1.33,0.35)**	**(-1.32,0.32)**	**(-1.29,0.25)**	**(-1.28,0.20)**
	3	2.28 (1.38)	2.73 (0.98)	2.84 (0.83)	2.94 (0.71)	3.00 (0.63)	3.05 (0.55)	3.12 (0.50)	3.13 (0.46)	3.17 (0.45)	3.19 (0.43)	3.21 (0.44)	3.16 (0.43)
	5	1.53 (0.88)	1.69 (0.84)	1.73 (0.82)	1.79 (0.81)	1.83 (0.80)	1.89 (0.79)	1.96 (0.79)	2.02 (0.78)	2.09 (0.77)	2.14 (0.75)	2.24 (0.72)	2.08 (0.73)
	7	1.20 (0.51)	1.18 (0.43)	1.15 (0.39)	1.13 (0.36)	1.11 (0.32)	1.12 (0.32)	1.10 (0.30)	1.08 (0.28)	1.08 (0.28)	1.07 (0.25)	1.04 (0.19)	1.00 (0.09)
0.50	**(µ,σ)**	**(-0.68,3.16)**	**(-0.87,1.11)**	**(-0.84,0.90)**	**(-0.81,0.76)**	**(-0.78,0.66)**	**(-0.76,0.58)**	**(-0.74,0.53)**	**(-0,72,0.48)**	**(-0.71,0.44)**	**(-0.70,0.40)**	**(-0.66,0.31)**	**(-0.64,0.25)**
	3	1.72 (1.08)	5.86 (2.80)	6.33 (2.59)	6.75 (2.45)	7.12 (2.34)	7.35 (2.22)	7.50 (2.10)	7.53 (2.01)	7.33 (1.92)	6.98 (1.81)	5.66 (1.48)	4.65 (1.09)
	5	1.21 (0.54)	4.06 (1.93)	4.36 (1.67)	4.62 (1.49)	4.87 (1.37)	4.95 (1.22)	4.96 (1.14)	4.80 (1.10)	4.53 (1.04)	4.21 (0.99)	3.29 (0.83)	2.75 (0.76)
	7	1.07 (0.28)	2.86 (1.43)	3.02 (1.29)	3.18 (1.14)	3.33 (1.04)	3.39 (0.91)	3.36 (0.85)	3.22 (0.83)	3.01 (0.84)	2.74 (0.84)	2.01 (0.80)	1.47 (0.60)
0.75	**(µ,σ)**	**(0.01,4.18)**	**(-0.42,1.30)**	**(-0.39,1.05)**	**(-0.36, 0.88)**	**(-0.34,0.77)**	**(-0.32, 0.68)**	**(-0.29, 0.61)**	**(-0.28,0.56)**	**(-0.26,0.51)**	**(-0.25,0.47)**	**(-0.21,0.37)**	**(-0.18,0.30)**
	3	1.33 (0.67)	13.03 (7.97)	20.28 (11.06)	26.22 (12.77)	28.78 (12.65)	25.75 (11.06)	20.08 (8.87)	15.76 (6.86)	12.63 (5.19)	10.51 (4.03)	7.05 (2.21)	5.48 (1.45)
	5	1.06 (0.28)	8.74 (4.98)	13.88 (7.29)	16.90 (7.90)	17.04 (7.11)	13.36 (5.40)	9.83 (3.75)	7.83 (2.75)	6.31 (2.02)	5.48 (1.60)	3.93 (1.01)	3.19 (0.81)
	7	1.01 (0.12)	6.10 (3.55)	10.15 (5.92)	12.44 (6.47)	10.65 (4.64)	7.99 (3.13)	5.92 (2.10)	4.84 (1.57)	4.02 (1.24)	3.49 (1.05)	2.50 (0.86)	1.90 (0.78)
1.0	**(µ,σ)**	**(0.61,5.00)**	**(-0.05,1.47)**	**(-0.03,1.18)**	**(0.00, 1.00)**	**(0.03,0.87)**	**(0.05,0.77)**	**(0.07,0.69)**	**(0.09,0.63)**	**(0.11,0.58)**	**(0.12,0.54)**	**(0.16,0.41)**	**(0.19,0.34)**
	3	1.18 (0.48)	12.92 (8.31)	47.26 (31.19)	370.73 (339.11)	99.20 (56.12)	47.65 (23.19)	30.73 (14.32)	22.36 (10.18)	17.54 (7.83)	14.37 (6.18)	8.18 (2.81)	6.28 (1.83)
	5	1.02 (0.16)	5.45 (1.59)	29.75 (17.79)	369.50 (358.50)	58.24 (30.41)	25.56 (12.06)	15.23 (6.73)	10.92 (4.39)	8.57 (3.20)	7.16 (2.39)	4.47 (1.18)	3.60 (0.90)
	7	1.00 (0.08)	5.07 (3.09)	19.56 (12.29)	369.33 (352.41)	35.36 (19.09)	15.02 (7.30)	8.96 (3.85)	6.54 (2.47)	5.25 (1.78)	4.46 (1.41)	2.89 (0.89)	2.28 (0.82)
1.50	**(µ,σ)**	**(1.67,6.26)**	**(0.58,1.86)**	**(0.58,1.44)**	**(0.61,1.21)**	**(0.64,1.05)**	**(0.66,0.94)**	**(0.68,0.84)**	**(0.70,0.77)**	**(0.72,0.71)**	**(0.74,0.65)**	**(0.79,0.51)**	**(0.82,0.41)**
	3	1.08 (0.30)	4.54 (2.94)	7.82 (4.71)	9.41 (5.25)	9.44 (4.84)	9.33 (4.36)	9.02 (3.87)	8.59 (3.39)	8.18 (2.99)	7.78 (2.65)	6.76 (1.77)	6.04 (1.32)
	5	1.00 (0.09)	2.88 (1.85)	5.32 (3.07)	6.58 (3.50)	6.44 (2.99)	6.32 (2.65)	6.03 (2.25)	5.77 (1.95)	5.46 (1.68)	5.22 (1.47)	4.54 (1.00)	3.97 (0.84)
	7	1.00 (0.02)	2.01 (1.25)	3.66 (2.18)	4.61 (2.52)	4.57 (2.23)	4.36 (1.93)	4.15 (1.58)	3.99 (1.40)	3.78 (1.21)	3.59 (1.07)	3.11 (0.78)	2.66 (0.75)
2.00	**(µ,σ)**	**(2.57,7.16)**	**(1.16,2.43)**	**(1.11,1.70)**	**(1.13,1.41)**	**(1.15,1.23)**	**(1.18,1.09)**	**(1.20,0.98)**	**(1.23,0.89)**	**(1.25,0.82)**	**(1.26,0.76)**	**(1.32,0.59)**	**(1.36,0.48)**
	3	1.05 (0.24)	2.22 (1.45)	3.49 (2.06)	3.98 (1.90)	3.88 (1.87)	3.79 (1.62)	3.69 (1.44)	3.57 (1.27)	3.51 (1.14)	3.44 (1.03)	3.25 (0.75)	3.11 (0.58)
	5	1.00 (0.07)	1.48 (0.84)	2.32 (1.44)	2.66 (1.50)	2.64 (1.40)	2.54 (1.25)	2.49 (1.16)	2.37 (1.08)	2.34 (1.02)	2.31 (0.98)	2.10 (0.86)	1.97 (0.81)
	7	1.00 (0.01)	1.25 (0.58)	1.67 (0.97)	1.55 (0.82)	1.8 (1.03)	1.75 (0.91)	1.70 (0.84)	1.58 (0.76)	1.51 (0.71)	1.45 (0.65)	1.27 (0.49)	1.13 (0.35)
3.00	**(µ,σ)**	**(4.00,8.33)**	**(2.35,4.00)**	**(2.08,2.58)**	**(2.03,1.86)**	**(2.04, 1.55)**	**(2.07, 1.37)**	**(2.10, 1.24)**	**(2.12, 1.13)**	**(2.15, 1.04)**	**(2.17, 0.96)**	**(2.24, 0.75)**	**(2.29, 0.61)**
	3	**1.02** **(0.17)**	1.22 (0.53)	1.58 (0.89)	1.76 (0.97)	1.76 (0.91)	1.71 (0.83)	1.65 (0.77)	1.62 (0.73)	1.55 (0.67)	1.51 (0.63)	1.47 (0.57)	1.27 (0.45)
	5	1.00 (0.04)	1.04 (0.22)	1.15 (0.44)	1.24 (0.56)	1.22 (0.52)	1.18 (0.45)	1.13 (0.39)	1.09 (0.32)	1.07 (0.28)	1.05 (0.22)	1.00 (0.09)	1.00 (0.03)
	7	1.00 (0.01)	1.00 (0.08)	1.04 (0.22)	1.07 (0.28)	1.06 (0.26)	1.04 (0.21)	1.02 (0.15)	1.01 (0.10)	1.00 (0.07)	1.00 (0.05)	1.00 (0.00)	1.00 (0.00)
4.00	**(µ,σ)**	**(5.12,9.07)**	**(3.61,5.53)**	**(3.15,3.99)**	**(2.93,2.81)**	**(2.86, 2.07)**	**(2.86, 1.68)**	**(2.88, 1.48)**	**(2.91, 1.35)**	**(2.94, 1.24)**	**(2.96, 1.15)**	**(3.04, 0.89)**	**(3.10, 0.73)**
	3	1.02 (0.14)	1.07 (0.28)	1.14 (0.42)	1.25 (0.55)	1.27 (0.55)	1.23 (0.49)	1.18 (0.42)	1.14 (0.37)	1.12 (0.34)	1.09 (0.30)	1.03 (0.17)	1.00 (0.09)
	5	1.00 (0.02)	1.00 (0.08)	1.01 (0.13)	1.04 (0.21)	1.04 (0.22)	1.02 (0.16)	1.01 (0.12)	1.00 (0.08)	1.00 (0.06)	1.00 (0.04)	1.00 (0.01)	1.00 (0.00)
	7	1.00 (0.00)	1.00 (0.02)	1.00 (0.05)	1.00 (0.00)	1.00 (0.08)	1.00 (0.05)	1.00 (0.03)	1.00 (0.01)	1.00 (0.01)	1.00 (0.00)	1.00 (0.00)	1.00 (0.00)

**Table 3:** ARL and SDRL results of Bayesian CC applying *W(1,1.5)* with $\gamma$=0.10 with subgroup size as 3, 5, and 7 under LLF.

*α*	*n*	Shape pameter = *λ*											
		0.50	1.00	1.25	1.50	1.75	2.00	2.25	2.50	2.75	3.00	4.00	5.00
		**ARL** **(SDRL)**	**ARL** **(SDRL)**	**ARL** **(SDRL)**	**ARL** **(SDRL)**	**ARL** **(SDRL)**	**ARL** **(SDRL)**	**ARL** **(SDRL)**	**ARL** **(SDRL)**	**ARL** **(SDRL)**	**ARL** **(SDRL)**	**ARL** **(SDRL)**	**ARL** **(SDRL)**
0.25	**(µ,σ)**	**(-1.51,2.04)**	**(-1.49,0.89)**	**(-1.45,0.72)**	**(-1.42,0.61)**	**(-1.40,0.53)**	**(-1.38,0.47)**	**(-1.36,0.42)**	**(-1.35,0.38)**	**(-1.33,0.35)**	**(-1.32,0.32)**	**(-1.29,0.25)**	**(-1.28,0.20)**
	3	1.69 (0.92)	1.85 (0.67)	1.89 (0.57)	1.92 (0.51)	1.96 (0.43)	1.98 (0.38)	2.00 (0.32)	2.00 (0.23)	2.01 (0.26)	2.01 (0.22)	2.00 (0.20)	1.97 (0.17)
	5	1.36 (0.58)	1.45 (0.52)	1.51 (0.51)	1.54 (0.50)	1.58 (0.49)	1.61 (0.48)	1.67 (0.46)	1.70 (0.45)	1.75 (0.43)	1.77 (0.42)	1.82 (0.38)	1.75 (0.42)
	7	1.17 (0.40)	1.17 (0.38)	1.14 (0.35)	1.13 (0.33)	1.12 (0.32)	1.06 (0.24)	1.10 (0.30)	1.08 (0.27)	1.08 (0.28)	1.06 (0.25)	1.03 (0.19)	1.00 (0.09)
0.50	**(µ,σ)**	**(-0.68,3.16)**	**(-0.87,1.11)**	**(-0.84,0.90)**	**(-0.81,0.76)**	**(-0.78,0.66)**	**(-0.76,0.58)**	**(-0.74,0.53)**	**(-0.72,0.48)**	**(-0.71,0.44)**	**(-0.70,0.40)**	**(-0.66,0.31)**	**(-0.64,0.25)**
	3	1.41 (0.72)	3.71 (2.18)	3.83 (1.96)	3.98 (1.82)	4.13 (1.72)	4.13 (1.56)	4.27 (1.53)	4.12 (1.40)	4.02 (1.29)	3.85 (1.20)	3.32 (1.00)	2.82 (0.78)
	5	1.16 (0.40)	2.77 (1.33)	2.83 (1.14)	2.89 (1.02)	2.99 (0.95)	2.95 (0.84)	2.91 (0.77)	2.80 (0.71)	2.73 (0.68)	2.59 (0.64)	2.17 (0.50)	1.92 (0.38)
	7	1.06 (0.25)	2.25 (1.03)	2.32 (0.92)	2.40 (0.82)	2.46 (0.75)	2.45 (0.67)	2.41 (0.64)	2.33 (0.61)	2.20 (0.58)	2.05 (0.54)	1.69 (0.49)	1.40 (0.49)
0.75	**(µ,σ)**	**(0.01,4.18)**	**(-0.42,1.30)**	**(-0.39,1.05)**	**(-0.36, 0.88)**	**(-0.34,0.77)**	**(-0.32, 0.68)**	**(-0.29, 0.61)**	**(-0.28,0.56)**	**(-0.26,0.51)**	**(-0.25,0.47)**	**(-0.21,0.37)**	**(-0.18,0.30)**
	3	1.19 (0.46)	8.11 (7.30)	13.43 (10.99)	16.83 (12.26)	17.05 (11.38)	14.48 (9.13)	11.40 (6.90)	9.21 (5.30)	7.34 (3.93)	6.18 (3.05)	4.16 (1.63)	3.29 (1.05)
	5	1.06 (0.25)	5.39 (3.91)	8.86 (6.47)	10.26 (6.72)	9.45 (5.56)	7.35 (3.84)	5.69 (2.65)	4.65 (1.98)	3.85 (1.45)	3.39 (1.15)	2.51 (0.67)	2.12 (0.48)
	7	1.01 (0.12)	4.25 (2.76)	6.88 (4.50)	7.78 (4.65)	6.86 (3.57)	5.37 (2.40)	2.40 (0.63)	3.50 (1.25)	2.93 (0.96)	2.58 (0.79)	1.96 (0.55)	1.64 (0.49)
1.0	**(µ,σ)**	**(0.61,5.00)**	**(-0.05,1.47)**	**(-0.03,1.18)**	**(0.00, 1.00)**	**(0.03,0.87)**	**(0.05,0.77)**	**(0.07,0.69)**	**(0.09,0.63)**	**(0.11,0.58)**	**(0.12,0.54)**	**(0.16,0.41)**	**(0.19,0.34)**
	3	1.09 (0.32)	7.95 (7.57)	36.45 (35.72)	369.03 (397.77)	87.22 (62.04)	36.79 (25.49)	20.22 (13.65)	13.92 (8.90)	10.47 (6.29)	8.51 (4.81)	4.81 (2.08)	3.76 (1.35)
	5	1.03 (0.17)	4.87 (3.61)	21.91 (19.99)	369.67 (394.21)	47.09 (33.45)	16.89 (11.49)	9.33 (5.47)	6.54 (3.40)	5.14 (2.37)	4.32 (1.75)	2.81 (0.83)	2.33 (0.59)
	7	1.00 (0.07)	3.79 (2.50)	15.99 (13.59)	370.86 (392.00)	33.10 (23.17)	11.43 (7.22)	6.46 (3.31)	4.65 (2.03)	3.74 (1.44)	3.25 (1.12)	2.18 (0.62)	1.82 (0.50)
1.50	**(µ,σ)**	**(1.67,6.26)**	**(0.58,1.86)**	**(0.58,1.44)**	**(0.61,1.21)**	**(0.64,1.05)**	**(0.66,0.94)**	**(0.68,0.84)**	**(0.70,0.77)**	**(0.72,0.71)**	**(0.74,0.65)**	**(0.79,0.51)**	**(0.82,0.41)**
	3	1.05 (0.25)	2.84 (2.08)	4.75 (3.85)	5.77 (4.42)	5.88 (4.03)	5.69 (3.48)	5.35 (2.99)	5.00 (2.53)	4.77 (2.24)	4.45 (1.89)	3.78 (1.22)	3.39 (0.91)
	5	1.01 (0.10)	2.12 (1.16)	3.39 (2.14)	4.13 (2.56)	4.13 (2.26)	3.90 (1.89)	3.70 (1.65)	3.50 (1.39)	3.33 (1.20)	3.14 (1.00)	2.73 (0.69)	2.42 (0.56)
	7	1.00 (0.02)	1.72 (0.89)	2.78 (1.57)	3.35 (1.88)	3.33 (1.67)	3.20 (1.42)	3.03 (1.22)	2.88 (1.04)	2.75 (0.92)	2.60 (0.79)	2.25 (0.57)	1.97 (0.46)
2.00	**(µ,σ)**	**(2.57,7.16)**	**(1.16,2.43)**	**(1.11,1.70)**	**(1.13,1.41)**	**(1.15,1.23)**	**(1.18,1.09)**	**(1.20,0.98)**	**(1.23,0.89)**	**(1.25,0.82)**	**(1.26,0.76)**	**(1.32,0.59)**	**(1.36,0.48)**
	3	1.03 (0.18)	1.68 (0.97)	2.30 (1.39)	2.52 (1.45)	2.57 (1.34)	2.48 (1.16)	2.40 (1.03)	2.31 (0.90)	2.25 (0.81)	2.21 (0.74)	2.08 (0.53)	2.00 (0.40)
	5	2.42 (0.56)	1.34 (0.59)	1.81 (0.90)	2.03 (0.92)	2.00 (0.88)	1.93 (0.78)	1.73 (0.63)	1.82 (0.61)	1.78 (0.58)	1.77 (0.54)	1.69 (0.48)	1.65 (0.471.00)
	7												
3.00	**(µ,σ)**	**(4.00,8.33)**	**(2.35,4.00)**	**(2.08,2.58)**	**(2.03,1.86)**	**(2.04, 1.55)**	**(2.07, 1.37)**	**(2.10, 1.24)**	**(2.12, 1.13)**	**(2.15, 1.04)**	**(2.17, 0.96)**	**(2.24, 0.75)**	**(2.29, 0.61)**
	3	1.01 (0.13)	1.13 (0.37)	1.31 (0.59)	1.42 (0.63)	1.41 (0.60)	1.37 (0.56)	1.33 (0.51)	1.31 (0.49)	1.27 (0.46)	1.23 (0.43)	1.14 (0.35)	1.06 (0.25)
	5	1.00 (0.04)	1.03 (0.19)	1.13 (0.36)	1.19 (0.42)	1.18 (0.40)	1.15 (0.36)	1.11 (0.32)	1.09 (0.29)	1.06 (0.25)	1.04 (0.21)	1.01 (0.10)	1.00 (0.03)
	7												
4.00	**(µ,σ)**	**(5.12,9.07)**	**(3.61,5.53)**	**(3.15,3.99)**	**(2.93,2.81)**	**(2.86, 2.07)**	**(2.86, 1.68)**	**(2.88, 1.48)**	**(2.91, 1.35)**	**(2.94, 1.24)**	**(2.96, 1.15)**	**(3.04, 0.89)**	**(3.10, 0.73)**
	3	1.01 (0.12)	1.05 (0.23)	1.09 (0.31)	1.14 (0.39)	1.14 (0.37)	1.12 (0.34)	1.09 (0.29)	1.07 (0.25)	1.04 (0.21)	1.03 (0.19)	1.00 (0.07)	1.00 (0.03)
	5	1.00 (0.02)	1.00 (0.08)	1.02 (0.14)	1.06 (0.26)	1.04 (0.20)	1.02 (0.16)	1.01 (0.12)	1.00 (0.08)	1.00 (0.06)	1.00 (0.03)	1.00 (0.01)	1.00 (0.0)
	7	1.00 (0.00)	1.00 (0.02)	1.00 (0.06)	1.00 (0.09)	1.00 (0.07)	1.00 (0.05)	1.00 (0.02)	1.00 (0.01)	1.00 (0.00)	1.00 (0.00)	1.00 (0.00)	1.00 (0.00)

**Table 4:** Run length outcomes of suggested CC with *W(1,1.5)* with $\gamma$=0.25 and considering size as 3, 5, and 7 under LLF.

*α*	*n*	Shape parameter = *λ*											
		0.50	1.00	1.25	1.50	1.75	2.00	2.25	2.50	2.75	3.00	4.00	5.00
		**ARL** **(SDRL)**	**ARL** **(SDRL)**	**ARL** **(SDRL)**	**ARL** **(SDRL)**	**ARL** **(SDRL)**	**ARL** **(SDRL)**	**ARL** **(SDRL)**	**ARL** **(SDRL)**	**ARL** **(SDRL)**	**ARL** **(SDRL)**	**ARL** **(SDRL)**	**ARL** **(SDRL)**
0.25	**(µ,σ)**	**(-1.51,2.04)**	**(-1.49,0.89)**	**(-1.45,0.72)**	**(-1.42,0.61)**	**(-1.40,0.53)**	**(-1.38,0.47)**	**(-1.36,0.42)**	**(-1.35,0.38)**	**(-1.33,0.35)**	**(-1.32,0.32)**	**(-1.29,0.25)**	**(-1.28,0.20)**
	3	2.25 (1.36)	2.71 (0.99)	2.83 (0.90)	2.93 (0.72)	2.99 (0.63)	3.05 (0.55)	3.10 (0.50)	3.12 (0.46)	3.16 (0.44)	3.17 (0.42)	3.20 (0.43)	3.15 (0.42)
	5	1.52 (0.88)	1.69 (0.83)	1.73 (0.82)	1.77 (0.81)	1.85 (0.80)	1.90 (0.80)	1.97 (0.80)	2.02 (0.78)	2.09 (0.79)	2.16 (0.75)	2.24 (0.74)	2.39 (0.75)
	7	1.22 (0.55)	1.21 (0.50)	1.18 (0.46)	1.15 (0.43)	1.12 (0.38)	1.12 (0.36)	1.11 (0.34)	1.09 (0.32)	1.08 (0.30)	1.07 (0.28)	1.17 (0.43)	1.01 (0.10)
0.50	**(µ,σ)**	**(-0.68,3.16)**	**(-0.87,1.11)**	**(-0.84,0.90)**	**(-0.81,0.76)**	**(-0.78,0.66)**	**(-0.76,0.58)**	**(-0.74,0.53)**	**(-0.72,0.48)**	**(-0.71,0.44)**	**(-0.70,0.40)**	**(-0.66,0.31)**	**(-0.64,0.25)**
	3	1.74 (1.06)	5.84 (2.82)	6.31 (2.58)	6.65 (2.39)	7.10 (2.34)	7.29 (2.16)	7.48 (2.13)	7.53 (2.01)	7.34 (1.90)	6.93 (1.80)	5.63 (1.46)	4.62 (1.08)
	5	1.22 (0.55)	4.08 (1.92)	4.40 (1.69)	4.64 (1.50)	4.87 (1.36)	4.96 (1.23)	4.95 (1.14)	4.82 (1.08)	4.89 (1.09)	4.22 (1.01)	3.30 (0.82)	2.76 (0.76)
	7	1.07 (0.29)	3.15 (1.62)	3.38 (1.47)	3.61 (1.31)	3.80 (1.17)	3.84 (1.04)	3.85 (0.99)	3.70 (0.95)	3.43 (0.95)	3.09 (0.96)	2.24 (0.93)	1.60 (0.79)
0.75	**(µ,σ)**	**(0.01,4.18)**	**(-0.42,1.30)**	**(-0.39,1.05)**	**(-0.36, 0.88)**	**(-0.34,0.77)**	**(-0.32, 0.68)**	**(-0.29, 0.61)**	**(-0.28,0.56)**	**(-0.26,0.51)**	**(-0.25,0.47)**	**(-0.21,0.37)**	**(-0.18,0.30)**
	3	1.33 (0.67)	12.84 (7.83)	20.20 (11.16)	26.20 (12.65)	28.37 (12.39)	25.72 (11.12)	19.86 (8.82)	15.89 (6.95)	12.55 (5.16)	10.52 (4.11)	6.96 (2.21)	5.45 (1.42)
	5	1.07 (0.28)	8.80 (4.98)	13.96 (7.25)	17.12 (7.94)	17.15 (7.15)	14.38 (6.17)	9.80 (3.71)	7.82 (2.73)	6.32 (1.99)	5.50 (1.59)	3.96 (1.01)	3.21 (0.82)
	7	1.01 (0.12)	6.85 (3.83)	10.86 (5.41)	12.84 (5.71)	12.46 (4.94)	9.44 (3.50)	6.86 (2.33)	5.56 (1.81)	4.59 (1.38)	3.98 (1.19)	2.82 (0.96)	2.12 (0.93)
1.0	**(µ,σ)**	**(0.61,5.00)**	**(-0.05,1.47)**	**(-0.03,1.18)**	**(0.00, 1.00)**	**(0.03,0.87)**	**(0.05,0.77)**	**(0.07,0.69)**	**(0.09,0.63)**	**(0.11,0.58)**	**(0.12,0.54)**	**(0.16,0.41)**	**(0.19,0.34)**
	3	1.19 (0.50)	12.48 (8.08)	47.02 (31.11)	369.10 (333.43)	97.29 (55.28)	47.78 (22.94)	30.51 (14.11)	22.29 (10.14)	17.52 (7.79)	14.49 (6.22)	8.13 (2.80)	6.27 (1.85)
	5	1.02 (0.17)	7.63 (4.64)	29.97 (18.42)	370.33 (335.84)	58.06 (30.06)	25.82 (11.99)	15.30 (6.84)	10.92 (4.41)	8.53 (3.16)	7.15 (2.42)	4.47 (1.20)	3.61 (0.90)
	7	1.00 (0.07)	5.76 (3.43)	22.77 (13.11)	370.36 (345.44)	42.70 (21.49)	17.98 (8.21)	10.52 (4.27)	7.62 (2.74)	6.05 (1.99)	5.12 (1.60)	3.24 (1.03)	2.53 (0.94)
1.50	**(µ,σ)**	**(1.67,6.26)**	**(0.58,1.86)**	**(0.58,1.44)**	**(0.61,1.21)**	**(0.64,1.05)**	**(0.66,0.94)**	**(0.68,0.84)**	**(0.70,0.77)**	**(0.72,0.71)**	**(0.74,0.65)**	**(0.79,0.51)**	**(0.82,0.41)**
	3	1.08 (0.31)	4.52 (2.90)	7.87 (4.68)	9.43 (5.29)	9.49 (4.82)	9.20 (4.38)	8.93 (3.82)	8.59 (3.46)	8.20 (3.03)	7.74 (2.62)	6.69 (1.71)	5.99 (1.28)
	5	1.00 (0.09)	2.90 (1.84)	5.39 (3.10)	6.53 (3.43)	6.50 (3.03)	6.37 (2.67)	6.06 (2.27)	5.82 (1.97)	5.51 (1.71)	5.25 (1.44)	4.56 (0.99)	3.99 (0.83)
	7	1.00 (0.03)	2.15 (1.40)	4.10 (2.43)	5.14 (2.69)	5.10 (2.41)	4.91 (2.04)	4.68 (1.76)	4.51 (1.56)	4.30 (1.37)	4.10 (1.20)	3.55 (0.91)	3.01 (0.89)
2.00	**(µ,σ)**	**(2.57,7.16)**	**(1.16,2.43)**	**(1.11,1.70)**	**(1.13,1.41)**	**(1.15,1.23)**	**(1.18,1.09)**	**(1.20,0.98)**	**(1.23,0.89)**	**(1.25,0.82)**	**(1.26,0.76)**	**(1.32,0.59)**	**(1.36,0.48)**
	3	1.05 (0.24)	2.22 (1.44)	3.45 (2.05)	3.80 (2.04)	3.87 (1.86)	3.77 (1.60)	3.67 (1.41)	3.55 (1.27)	3.49 (1.13)	3.45 (1.04)	3.22 (0.73)	3.09 (0.58)
	5	1.00 (0.06)	1.48 (0.84)	2.36 (1.46)	2.62 (1.50)	2.68 (1.42)	2.58 (1.28)	2.49 (1.18)	2.41 (1.08)	2.32 (1.02)	2.29 (0.98)	2.11 (0.87)	1.98 (0.78)
	7	1.00 (0.01)	1.20 (0.54)	1.77 (1.11)	1.99 (1.19)	1.98 (1.14)	1.88 (1.03)	1.80 (0.97)	1.69 (0.89)	1.62 (0.84)	1.55 (0.80)	1.33 (0.62)	1.15 (0.42)
3.00	**(µ,σ)**	**(4.00,8.33)**	**(2.35,4.00)**	**(2.08,2.58)**	**(2.03,1.86)**	**(2.04, 1.55)**	**(2.07, 1.37)**	**(2.10, 1.24)**	**(2.12, 1.13)**	**(2.15, 1.04)**	**(2.17, 0.96)**	**(2.24, 0.75)**	**(2.29, 0.61)**
	3	1.03 (0.18)	1.23 (0.54)	1.54 (0.88)	1.78 (0.98)	1.77 (0.90)	1.70 (0.82)	1.65 (0.77)	1.61 (0.72)	1.55 (0.67)	1.51 (0.64)	1.36 (0.52)	1.27 (0.45)
	5	1.00 (0.03)	1.03 (0.20)	1.15 (0.45)	1.23 (0.55)	1.22 (0.53)	1.18 (0.46)	1.13 (0.39)	1.09 (0.31)	1.06 (0.26)	1.04 (0.21)	1.00 (0.08)	1.00 (0.08)
	7	1.00 (0.00)	1.00 (0.08)	1.04 (0.22)	1.07 (0.30)	1.06 (0.27)	1.04 (0.21)	1.02 (0.15)	1.01 (0.12)	1.00 (0.07)	1.00 (0.05)	1.00 (0.01)	1.00 (0.00)
4.00	**(µ,σ)**	**(5.12,9.07)**	**(3.61,5.53)**	**(3.15,3.99)**	**(2.93,2.81)**	**(2.86, 2.07)**	**(2.86, 1.68)**	**(2.88, 1.48)**	**(2.91, 1.35)**	**(2.94, 1.24)**	**(2.96, 1.15)**	**(3.04, 0.89)**	**(3.10, 0.73)**
	3	1.01 (0.13)	1.07 (0.30)	1.15 (0.43)	1.25 (0.55)	1.27 (0.54)	1.22 (0.47)	1.18 (0.42)	1.15 (0.38)	1.11 (0.33)	1.09 (0.29)	1.03 (0.17)	1.00 (0.08)
	5	1.00 (0.02)	1.00 (0.09)	1.02 (0.15)	1.04 (0.22)	1.04 (0.22)	1.02 (0.16)	1.01 (0.11)	1.00 (0.08)	1.00 (0.05)	1.00 (0.04)	1.00 (0.00)	1.00 (0.00)
	7	1.00 (0.00)	1.00 (0.01)	1.00 (0.06)	1.01 (0.10)	1.00 (0.08)	1.00 (0.05)	1.00 (0.02)	1.00 (0.01)	1.00 (0.01)	1.00 (0.00)	1.00 (0.00)	1.00 (0.00)

## Real Life application

**Figure 1:**
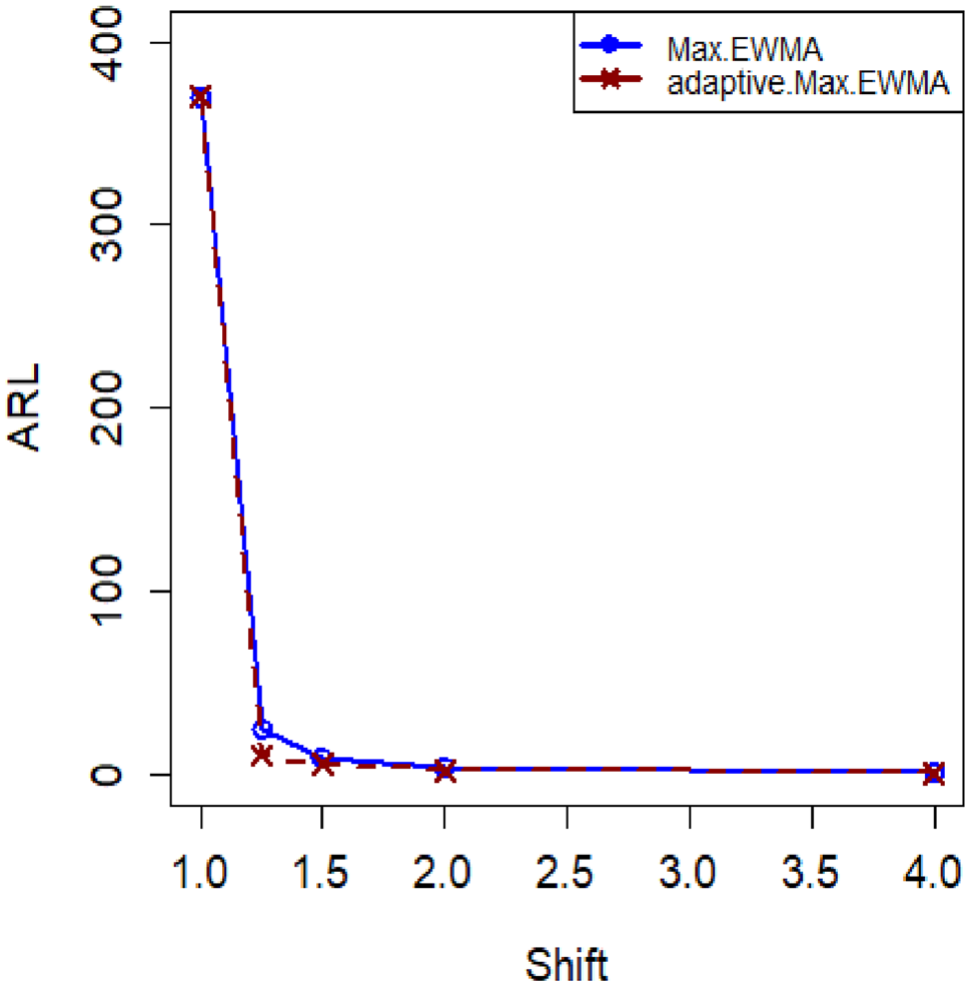
ARL plot for the offered CC under SELF.

**Figure 2:**
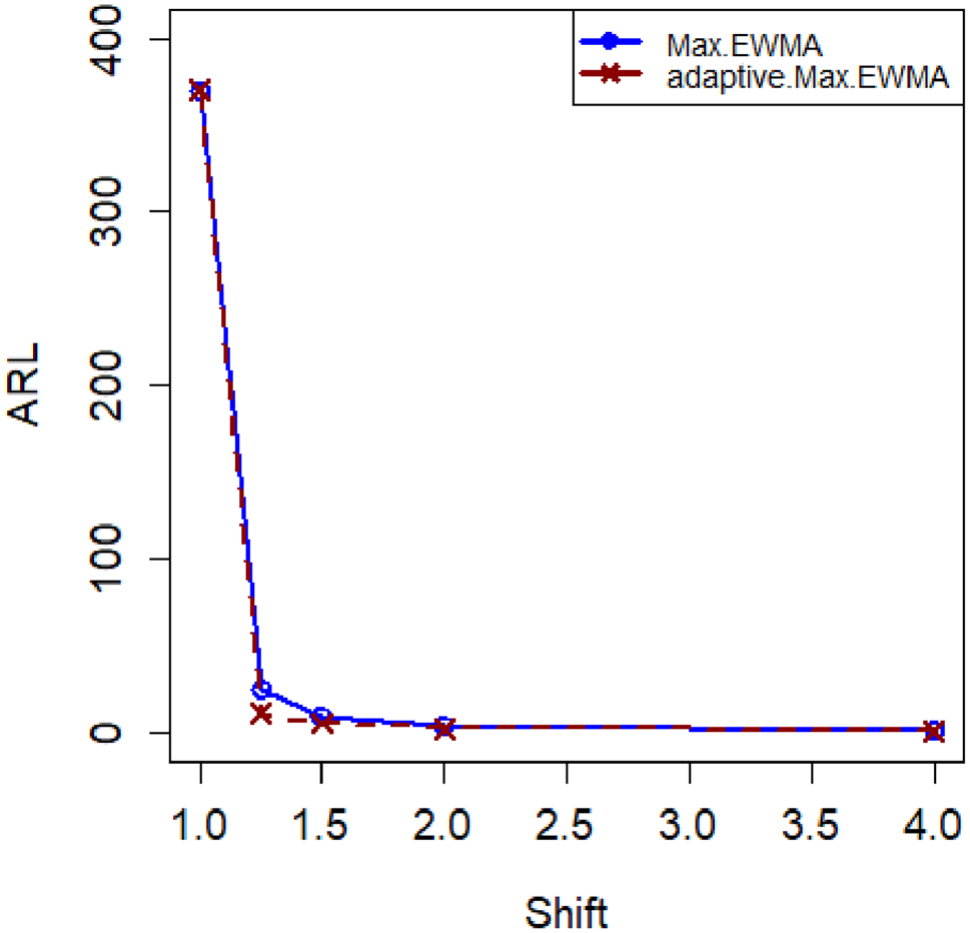
ARL plot for suggested CC utilizing LLF.

The article provides a practical illustration of the proposed CC by applying it to data obtained from Montgomery's research^27^, which focuses on the hard-bake process in semiconductor manufacturing. The dataset used in this application comprises 45 samples, with each sample containing 5 wafers, resulting in a total of 225 data points measured in microns. The measurements were taken at regular one-hour intervals during the data collection. Within the dataset, the initial 30 samples, totaling 150 observations, represent a well-controlled process referred to as the phase I dataset. This phase serves as a baseline for the controlled state of the manufacturing process. On the other hand, the subsequent 15 samples, comprising 75 observations, depict an out-of-control process and are identified as the phase II dataset. This phase is characterized by variations or deviations from the expected or controlled behavior in the semiconductor manufacturing process. The differentiation between phase I and phase II datasets allows for the assessment of the proposed CC's effectiveness in identifying and responding to variations, providing insights into its practical utility in monitoring and maintaining the quality of the manufacturing process (Figs. 1, 2).

**Figure 3:**
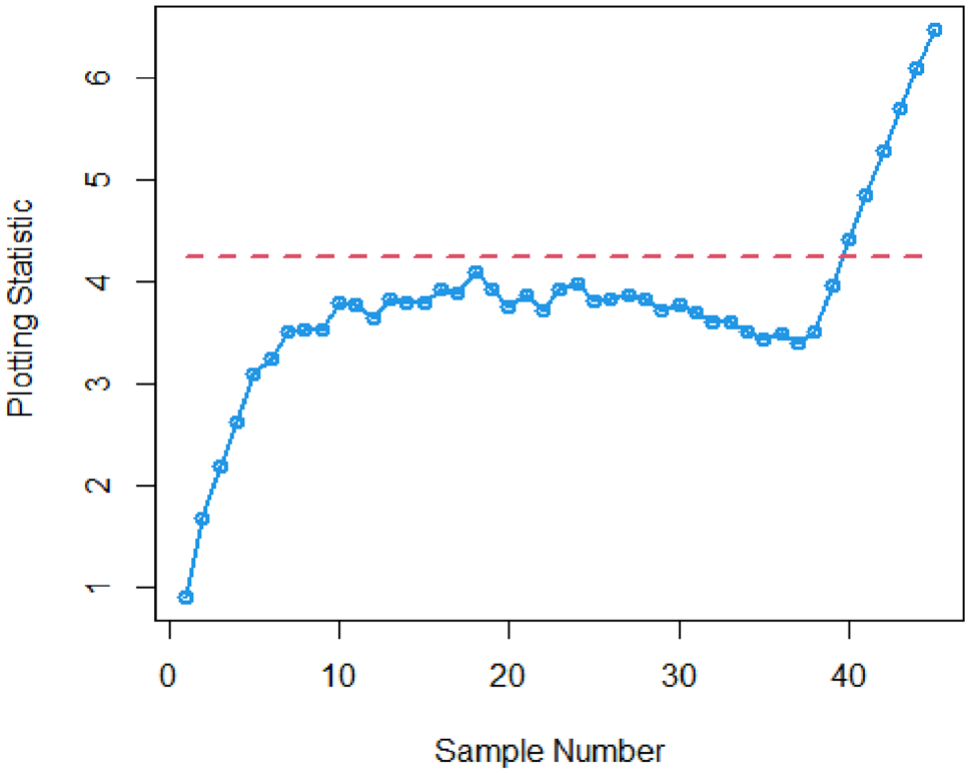
Existing Max-EWMA CC under Weibull process for jointly monitoring with $\lambda = 0.25$.

**Figure 4:**
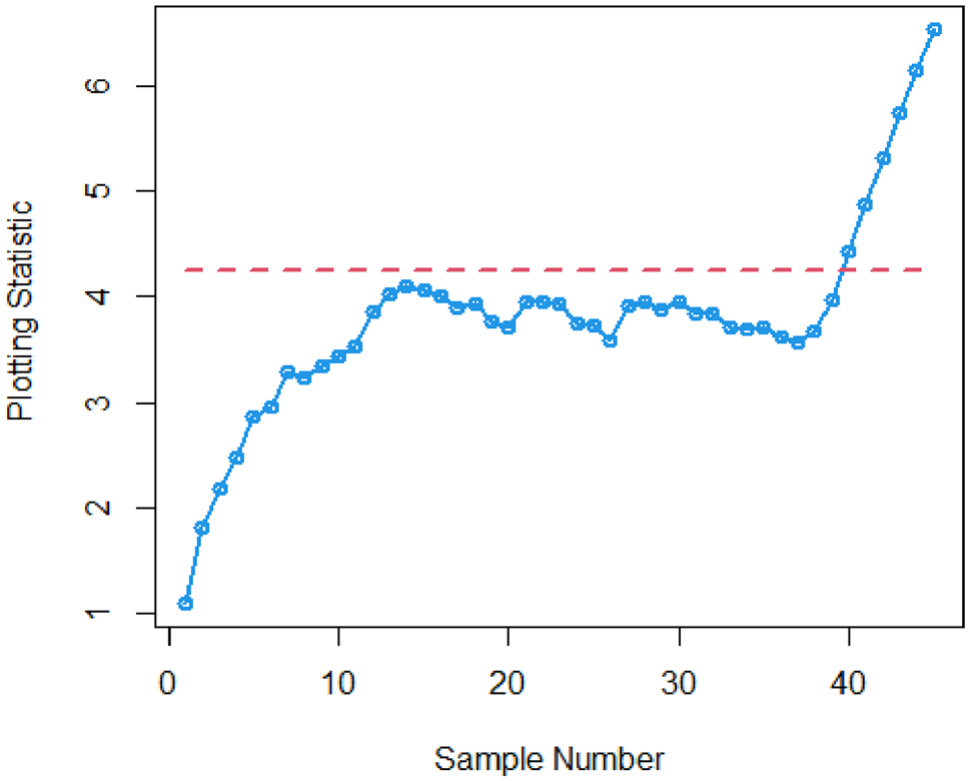
Utilizing LLF, Max-EWMA CC applying Weibull process for jointly monitoring at $\lambda = 0.25$.

**Figure 5:**
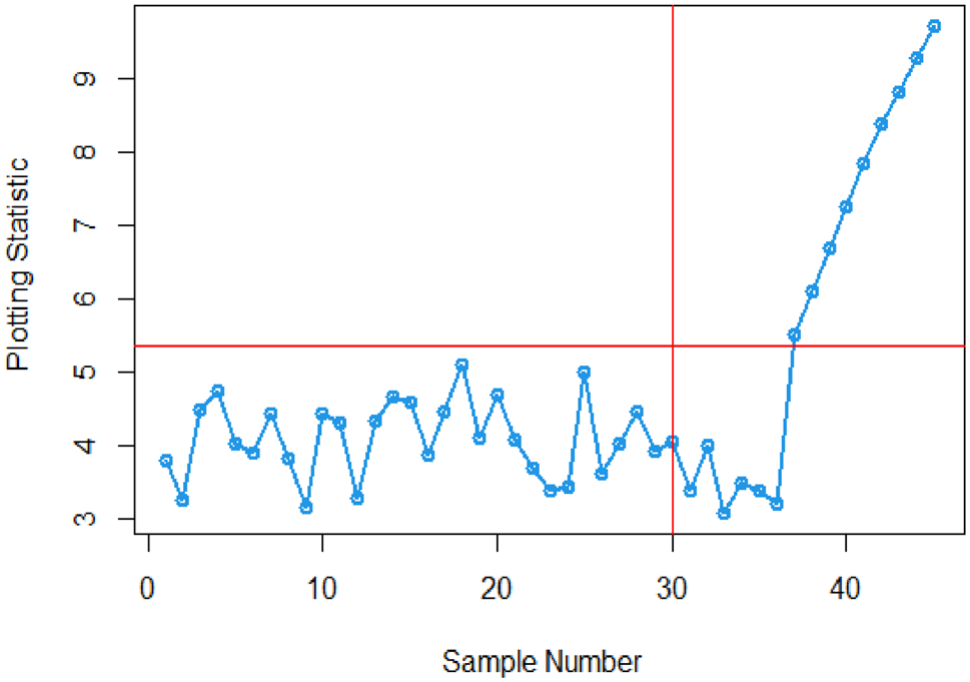
plot for the suggested CC Using SELF, for jointly monitoring with $\lambda = 0.25$.

**Figure 6:**
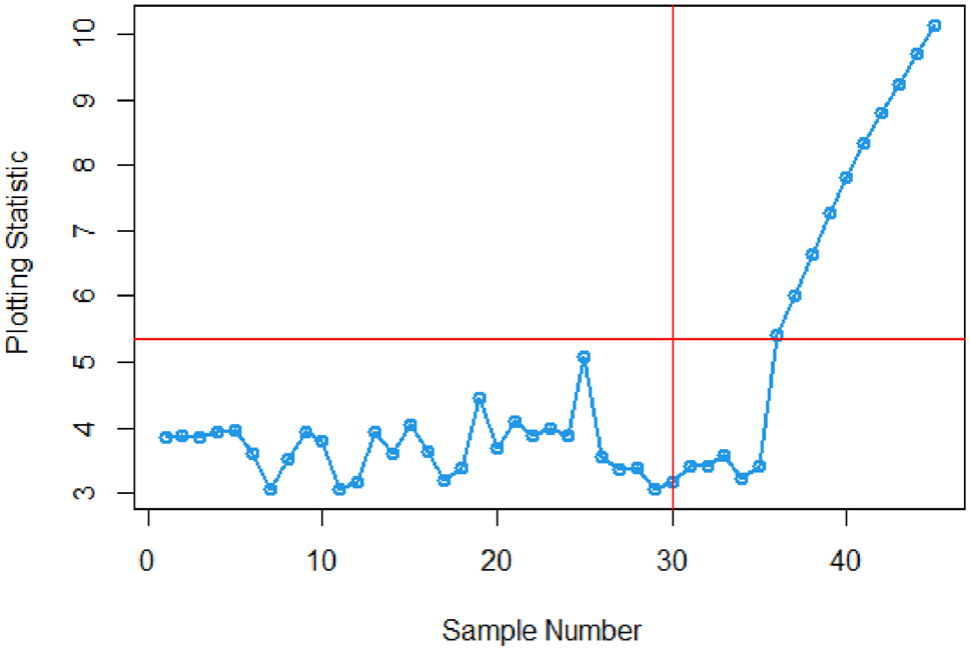
Plot using LLF, for recommended CC for jointly monitoring with $\lambda = 0.25$.

Figures 3 and 4 visually demonstrate the application of the existing Bayesian Max-EWMA CC, which is specifically designed to monitor both process mean and dispersion simultaneously, utilizing the SELF and LLF methods. A detailed examination of these charts clearly indicates instances where the process has deviated from the expected control state, notably in the 38th and 39th samples, particularly when considering a smoothing constant of 0.25, while Figs. 5 and 6 identifying the out-of-control signals for both the process mean and dispersion on the 35th and 36th samples for the proposed CC. These changes in process behavior are closely related to changes in process mean and dispersion of the Weibull distribution. In reliability and survival analysis, the Weibull distribution is a statistical model often used to describe the distribution of data. Changes in the shape and scale parameters directly influence the distribution's characteristics, resulting in the observed discrepancies in the control charts. In essence, the identified deviations in the 36th and 35th samples can be traced back to shifts in the fundamental parameters of the underlying statistical distribution, impacting the overall behavior of the manufacturing process and triggering the out-of-control conditions reflected in the CCs.

## Conclusion

In this research, we present an innovative Bayesian CC specifically designed for monitoring Weibull processes, enabling simultaneous tracking of process mean and variance. This CC includes informative prior distributions and integrates two different LFs within the framework of the posterior distribution. To thoroughly assess the performance of this novel approach, we conducted an extensive analysis, presenting detailed results in Tables 1, 2, 3 and 4 . ARL and SDRL were used as evaluation metrics These evaluations utilized key metrics such as ARL and SDRL and ARL plots i.e., Figs. 1 and 2 also show efficient performance of the suggested CC. A useful case study was also carried out, with an emphasis on the hard bake procedure used in the production of semiconductors. Importantly, when applied to posterior distributions, the proposed CC demonstrated significant proficiency in detecting out-of-control signals within the process. The insights gained from this study hold promise for extending these principles to the development of other memory-type Control Charts, enhancing their effectiveness across diverse industrial applications. Expanding the application of this innovative technique beyond non-normal distributions to various types of Control Charts contributes to a more comprehensive understanding of underlying data patterns. This increased effort reduces the likelihood of costly errors and defects by facilitating early detection of potential quality issues in multiple areas and enabling timely remedial action. This method is essential to quickly detect irregularities in patient data, enable rapid interventions, and improve patient care outcomes in real-world settings, particularly healthcare settings. Applying this methodology to non-normal distributions and various types of control charts in the manufacturing industry helps detect process variations, which in turn leads to improved product quality and waste reduction.

## Supplementary Information

Supplementary Information.

## Data Availability

If there is a reasonable request, interested individuals can directly obtain the datasets that were utilized and/or analyzed in the present study from the corresponding author.
