# Supplementary material for: Memory type Bayesian adaptive max-EWMA control chart for weibull processes
Source: Sci Rep. 2024 Apr 18;14:8923. doi: 10.1038/s41598-024-59680-6 (PMC11551196; doi:10.1038/s41598-024-59680-6)
Supplement: Supplementary file 1 — Supplementary Information. [file 41598_2024_59680_MOESM1_ESM.docx]

**Appendix**

**LIST OF ABBREVIATIONS**

| SQC | Statistical Quality Control |
| --- | --- |
| CC | Control Chart |
| EWMA | Exponentially Weighted Moving Average |
| CUSUM | Cumulative Sum |
| AEWMA | Adaptive Exponentially Weighted Moving Average |
| LF | Loss Function |
| SELF | Squared Error Loss Function |
| LLF | Linex Loss Function |
| ARL | Average Run Length |
| SDRL | Standard Deviation of Run Length |
| *ARLo* | ARL for in-control process |
| *ARL*1 | ARL for out-of-control process |
| *UCL* | Upper control limit |
| *LCL* | Lower control limit |
| SRS | Simple Random Sampling |
| $\lambda$ | Shape parameter |
| $\alpha$ | Scale parameter |
| $\gamma$ | Smoothing Constant |
| $\theta$ | Mean of normal distribution |
| $\delta^{2}$ | Variance of normal distribution |
| $\theta_{0}$ | Mean of prior distribution |
| $\delta_{0}^{2}$ | Variance of prior distribution |
| $\theta_{n}$ | Mean of posterior distribution |
| $\delta_{n}^{2}$ | Variance of posterior distribution |
| $\bar{x}$ | Mean of study variable obtain form SRS |
|  | Bayes estimator under SELF |
|  | Bayes estimator under LLF |
| $\delta_{1}^{2}$ | Variance for posterior predicative distribution |
| $\sigma$ | Shift |
| *h* | Thorold value |
| Var | Variance |
